# Structural Identification and Antioxidant Activity of Pine Nut Peptide–Zinc Chelate Complex

**DOI:** 10.3390/foods15020359

**Published:** 2026-01-19

**Authors:** Kexin Yang, Xiaotong Zhang, Jiayu Zhang, Zhi Zhang

**Affiliations:** 1 College of Forestry, Northeast Forestry University, Harbin 150040, China; zhangxiaotong9697@163.com; 2 College of Life Sciences, Northeast Forestry University, Harbin 150040, China; zjy15734693830@163.com

**Keywords:** pinus koraiensis, zinc supplement, structural identification, peptidomics, antioxidant capacity

## Abstract

To achieve the high-value utilization of pine nut resources, a novel zinc supplement was developed in this study. Pine nut protein was enzymatically hydrolyzed to prepare pine nut peptides (PP), which were subsequently chelated with zinc ions to form pine nut peptide–zinc chelate (PZn). Under optimized conditions, the zinc chelation rate of PZn reached 60.18 ± 1.77%. Peptidomic analysis revealed that PZn is composed of a select group of peptides predominantly characterized by low molecular weight (80.65 ± 1.47% < 1 kDa) and enrichment in aspartic acid, glutamic acid, and cysteine, indicating a self-selective chelation process. Comprehensive characterization via multiple techniques confirmed that zinc ions coordinate with carboxyl, hydroxyl, and thiol groups on these peptides, leading to charge neutralization, disruption of hydrogen-bonding networks, and peptide aggregation. Furthermore, bioactivity prediction of the PZn-constituting peptides revealed high intrinsic antioxidant potential, which corroborated the experimental results, showing that PZn exhibited significantly enhanced radical scavenging capacity compared to PP. These findings demonstrate that PZn possesses excellent zinc-binding capability and antioxidant activity, suggesting its potential as a novel zinc supplement, with its efficacy rooted in its specific molecular composition.

## 1. Introduction

Zinc is an essential trace element that plays a vital role in numerous physiological processes, including enzymatic catalysis, immune function, protein synthesis, and neurological development [1]. Deficiency in zinc can lead to impaired growth, weakened immunity, and cognitive disorders. Conventional zinc supplements, such as inorganic zinc salts, often suffer from low bioavailability and can cause gastrointestinal discomfort, highlighting the need for more efficient and tolerable alternatives [2].

Bioactive peptides derived from food proteins have gained significant attention for their ability to form stable complexes with metal ions. These peptide–metal chelates, characterized by their cyclic structures and thermodynamic stability, offer a promising solution for mineral supplementation [3]. Notably, peptide–zinc complexes demonstrate enhanced absorption and reduced side effects compared to their inorganic counterparts, as evidenced by improved solubility and gastrointestinal tolerance of soy protein hydrolysate zinc chelates [4,5]. This combination delivers both the nutritional benefits of zinc and the potential bioactivities of the peptides, such as antioxidant and immunomodulatory effects [6].

Pine nuts represent an excellent source of high-quality plant protein [7,8]. Their defatted meal contains over 65% protein (*w*/*w*) with a well-balanced amino acid profile that fulfills the FAO/WHO requirements for essential amino acids [9,10,11]. Recent studies have identified various bioactive peptides from pine nuts with demonstrated antioxidant, anti-fatigue, and immunomodulatory properties [12,13,14], underscoring their potential as a raw material for functional ingredient development. However, the potential of pine nut-derived peptides in forming zinc chelates remains largely unexplored.

This study aims to comprehensively investigate the preparation, characterization, and antioxidant activity of a zinc chelate complex derived from pine nut peptides (PZn). Specifically, we seek to: (1) employ peptidomic analysis to elucidate its molecular composition at the sequence level; (2) systematically characterize its structural properties using a multi-technique approach including UV, FT-IR, CD, Zeta potential, SEM, and EDS; and (3) evaluate its antioxidant capacity in comparison with unchelated pine nut peptides (PP) through in vitro radical scavenging assays. The findings will provide fundamental insights into the molecular mechanism of zinc-peptide chelation and support the development of a novel zinc supplement from underutilized pine nut resources.

## 2. Materials and Methods

### 2.1. Reagents

Defatted pine nut protein powder was provided by Mohe Wuxingjian Biotechnology Co., Ltd. (Mohe City, Heilongjiang, China). Alkaline protease (Alcalase^®^ 2.4 L, derived from *Bacillus licheniformis*, activity 4000 U/g, substrate: casein, assay conditions: pH 8.5, 50 °C, purity ≥ 98%) and ZnSO_4_·7H_2_O (purity ≥ 99%) were purchased from Biotopped^®^ Beijing Boaotuoda Technology Co., Ltd. (Beijing, China). All other chemicals were analytical grade from Shanghai Yuanye Bio-Technology Co., Ltd. (Shanghai, China).

### 2.2. Preparation of PP and PZn

A 4% (*w*/*v*) pine nut protein solution was prepared in deionized water. The pH was adjusted to 8.5 using 1 mol·L^−1^ NaOH, followed by enzymatic hydrolysis with alkaline protease (4000 U/g protein) at 50 °C for 2 h. The enzyme was inactivated by boiling for 15 min. After cooling to room temperature, the hydrolysate was centrifuged at 8000 rpm (4 °C) for 15 min using a high-speed refrigerated centrifuge (Sigma 3−18K, Munich, Germany). The supernatant was collected and freeze-dried to obtain PP.

PP was dissolved in deionized water (10 mg·mL^−1^). ZnSO_4_·7H_2_O was added at a peptide-to-zinc ratio of 1:1 (g: mmol). The pH was adjusted to 6.0 using 1 mol·L^−1^ NaOH (initial pH of PP solution was approximately 5.2–5.5). The mixture was incubated at 60 °C for 60 min with continuous shaking. After rapid cooling, four volumes of anhydrous ethanol (relative to the volume of the supernatant, *v*/*v*) were added for precipitation. The solution was stored at 4 °C for 1 h, then centrifuged at 8000 rpm for 15 min. The precipitate was washed repeatedly with 80% (*v*/*v*) ethanol to remove unbound zinc and freeze-dried to obtain PZn.

### 2.3. Determination of Zinc Chelating Ability

PZn was dissolved in deionized water (0.2 g·mL^−1^). A 10 mL aliquot was mixed with 10 mL NH_3_-NH_4_Cl buffer (pH 10). After adding chromazurol T indicator, the solution was titrated with 0.01 mol·L^−1^ Na_2_EDTA until color changed from purple to light blue. Zinc content was calculated as:

$$X=\left(M\times C\times V\right)/\left(m\times 1000\right)\times 100\%$$

where *X* is the zinc content in PZn (%); *M* is the atomic mass of zinc (g·moL^−1^); *C* is Na_2_EDTA concentration (mol·L^−1^); *V* is the volume of Na_2_EDTA consumed (mL); *m* is the mass of PZn (g). Methodology reference: GB 5009.14-2017 “National Food Safety Standard for the Determination of Zinc in Food” [15].

The chelation rate was calculated as:

$$Y=\left(X\times m/m^{\prime }\right)\times 100\%$$

where *Y* is the zinc chelation rate (%); *m* is the mass of PZn (g); *m’* is the actual mass of ZnSO_4_·7H_2_O added (g).

### 2.4. Peptidomic Analysis of PZn

The molecular composition of PZn was characterized by liquid chromatography-tandem mass spectrometry (LC-MS/MS). Briefly, PZn samples were dissolved in 0.1% formic acid (1 mg·mL^−1^), desalted using Millipore C18 ZipTips (Sigma-Aldrich, St. Louis, MO, USA), and analyzed on an Easy-nLC 1200 system coupled to a QExactive HF mass spectrometer (Thermo Fisher Scientific, Waltham, MA, USA). Chromatographic conditions: Acclaim PepMap RSLC C18 column (75 μm × 25 cm, 2 μm, 100 Å); mobile phase A (0.1% formic acid in water), mobile phase B (0.1% formic acid in acetonitrile); gradient program: 0–5 min 5% B, 5–40 min 5–35% B, 40–45 min 35–95% B, 45–50 min 95% B; flow rate 300 nL·min^−1^. Mass spectrometry conditions: data-dependent acquisition mode; full scan resolution 60,000 (*m*/*z* 350–1800); MS/MS resolution 15,000 (*m*/*z* 100–2000); fragmentation energy 27 eV; dynamic exclusion 30 s. The acquired raw data were processed using Byonic 5.10 software against the uniprotkb_taxonomy_id_3337_2025_10_21.fasta with precursor mass tolerance 10 ppm, fragment mass tolerance 0.02 Da, and FDR threshold <1%. Molecular weight distribution was calculated based on the identified sequences. Bioinformatic analysis for stability, allergenicity, toxicity, and bioactivity prediction was conducted using the BIOPEP-UWM database.

### 2.5. Amino Acid Composition Analysis

Analysis was performed according to GB 5009.124-2016 “National Food Safety Standard for the Determination of Amino Acids in Food” [16]. Briefly, samples were hydrolyzed with 6 mol·L^−1^ HCl at 110 °C for 24 h, then analyzed using an L-8900 amino acid analyzer (Hitachi, Tokyo, Japan).

### 2.6. Zeta Potential Measurement

Samples were dispersed in deionized water (1 mg·mL^−1^). Zeta potential was measured using a Zetasizer Nano S90 (Malvern, Nottingham, UK) at 25 °C after 2 min equilibration.

### 2.7. UV–vis Spectroscopy

PP and PZn solutions (0.1 mg·mL^−1^) were scanned from 200 to 400 nm using a UV–visible spectrophotometer (Shimadzu UV-2600, Kyoto, Japan).

### 2.8. FT-IR Spectroscopy

Samples (2 mg) were mixed with 200 mg dried KBr and pressed into transparent pellets. Spectra were recorded on an FT-IR spectrometer (Shimadzu IRAffinity-1s, Kyoto, Japan) from 4000 to 400 cm^−1^.

### 2.9. Circular Dichroism (CD) Spectroscopy

PP and PZn solutions (1 mg·mL^−1^) were analyzed using a JASCO J-1500 spectropolarimeter (Hitachi, Tokyo, Japan) from 190 to 250 nm. Spectra were converted to mean residue ellipticity [θ] and secondary structure content was calculated using CDProWin7 software.

### 2.10. SEM and EDS Spectroscopy

Samples were gold-sputtered (thickness ~10 nm) and observed under SEM (Hitachi SU8010, Tokyo, Japan) at 5 kV and an amplification factor of 5 k to 20 k. EDS analysis: Elemental composition was analyzed by EDS (attached to Hitachi SU8010 SEM) with acceleration voltage 5 kV, acquisition time 60 s, and element detection range B-U.

### 2.11. Antioxidant Activity Assays

The DPPH radical scavenging activities of PP and PZn were evaluated at various concentrations (1–6 mg·mL^−1^) according to the method described by Brand-Williams et al. (1995) with modifications [7,12,17]. Briefly, 2 mL of sample solution was mixed with 2 mL of fresh DPPH working solution (0.2 mmol·L^−1^ in 95% ethanol). The mixture was vortexed, incubated in the dark for 30 min, and the absorbance was measured at 517 nm. The scavenging activity was calculated as:

$$\mathrm{Scavenging}\;\mathrm{rate}\;(\%)=\left(1-\left(A_{1}-A_{2}\right)/A_{0}\right)\times 100\%$$

where *A*_1_ is the absorbance of the test group (sample + DPPH); *A_2_* is the absorbance of the sample background control (sample + ethanol); *A*_0_ is the absorbance of the DPPH blank control (water + DPPH).

The hydroxyl radical scavenging capacity of PP and PZn was evaluated at various concentrations (1–6 mg·mL^−1^). The reaction mixture contained 1 mL of FeSO_4_ (9 mmol·L^−1^), 1 mL of ethanolic salicylic acid (9 mmol·L^−1^, dissolved in 50% ethanol), 1 mL of sample solution, and 1 mL of H_2_O_2_ (8.8 mmol·L^−1^). A blank control (replacing sample with deionized water) and a sample background control (replacing H_2_O_2_ with deionized water) were set up. After incubation at 37 °C for 30 min in the dark, the absorbance was measured at 510 nm. The scavenging activity was calculated as:

$$\mathrm{Scavenging}\;\mathrm{rate}\;(\%)=\left(1-\left(A_{X}-A_{X0}\right)/A_{0}\right)\times 100\%$$

where *A_X_* is the absorbance of the sample with H_2_O_2_, *A_X_*_0_ is the absorbance of the sample background control (without H_2_O_2_), and *A*_0_ is he absorbance of the reagent blank control (H_2_O_2_ without sample).

All absorbance measurements were conducted using a UV–visible spectrophotometer (Shimadzu UV-2600, Kyoto, Japan) with a 1 cm quartz cuvette. The instrument was calibrated with blank solvent (ethanol for DPPH assay, deionized water for hydroxyl radical assay) before measurements.

The IC_50_ values of PP and PZn were not calculated, as the core objective of this study was to compare the antioxidant activity differences before and after pine nut peptide chelation with zinc ions (PP vs. PZn) rather than determining the half-maximal effective concentration of each sample individually. We focused on DPPH and hydroxyl radical scavenging assays because these radicals are closely related to the in vivo antioxidant effects of zinc supplements, while ABTS/FRAP/ORAC assays will be included in future studies.

### 2.12. Data Analysis

Excel 2022 was used for data collation, and Origin 8.5 was employed for graph generation. Statistical analysis was conducted using SPSS 25.0 software. An independent samples *t*-test was used to compare the significant differences between PP and PZn groups (e.g., amino acid composition, zeta potential, antioxidant activity). All experiments were performed in triplicate (*n* = 3), and data are presented as mean ± standard deviation (SD). Statistical significance was defined as *p* < 0.05 (*), and highly significant difference as *p* < 0.01 (**). Outliers were identified using the Grubbs test and excluded from analysis (α = 0.05).

## 3. Results and Discussion

### 3.1. Zinc Chelating Capacity

Under optimized conditions (peptide–zinc = 1:1 g:mmol, pH 6, 60 °C, 60 min), PZn achieved a chelation rate of 60.18 ± 1.77%. This efficiency is comparable to other plant peptide–zinc chelates reported previously. For instance, Fan et al. [18] reported strong metal-binding capabilities in walnut peptides, while Zhang et al. [19] isolated a specific pentapeptide–zinc chelate from almonds with substantial zinc content. The variation in chelating capacity among different peptide sources can be attributed to factors such as peptide sequence, acidic amino acid content, and molecular weight distribution [20].

### 3.2. Molecular Characteristics and Bioactivity Potential of PZn

#### 3.2.1. Molecular Weight Distribution

The molecular weight profile of the peptides constituting the PZn complex provides critical insights into the chelation mechanism. As detailed in Table 1, an overwhelming majority (80.65 ± 1.47%) of the identified peptides have a molecular weight below 1 kDa, with the entire population falling under 5 kDa.

This highly skewed distribution is not random but signifies a self-selective chelation process. Low molecular weight peptides possess greater conformational flexibility and a higher density of terminal functional groups per unit mass, which facilitates the formation of stable coordination bonds with Zn^2+^. The preference of Zn^2+^ for low-molecular-weight peptides (<1 kDa) can be attributed to two key factors: (1) steric hindrance: small peptides have higher conformational flexibility, allowing Zn^2+^ to easily access the coordination sites (carboxyl, thiol groups); (2) electrostatic interaction: low-molecular-weight peptides have a higher density of negative charges per unit mass, enhancing the attraction to cationic Zn^2+^ [21]. Consistent with this, we measured the chelation rate of high-molecular-weight peptides (>1 kDa) isolated from PP, which was only 12.30 ± 0.45%, further confirming that low-molecular-weight peptides are the main binders of Zn^2+^. Furthermore, the small size of these constituent peptides suggests favorable bioavailability potential for the PZn complex, as short peptides are generally more amenable to intestinal absorption.

The molecular weight distribution of PZn peptides is consistent with previous studies on walnut [18] and almond [19] peptides, where >70% of the peptides involved in metal chelation were <1 kDa. This indicates that the preference for low-molecular-weight peptides is a common feature of plant peptide–metal chelates.

#### 3.2.2. N– and C–Terminal Amino Acid Propensity and Zinc Binding Sites

**Figure 1:**
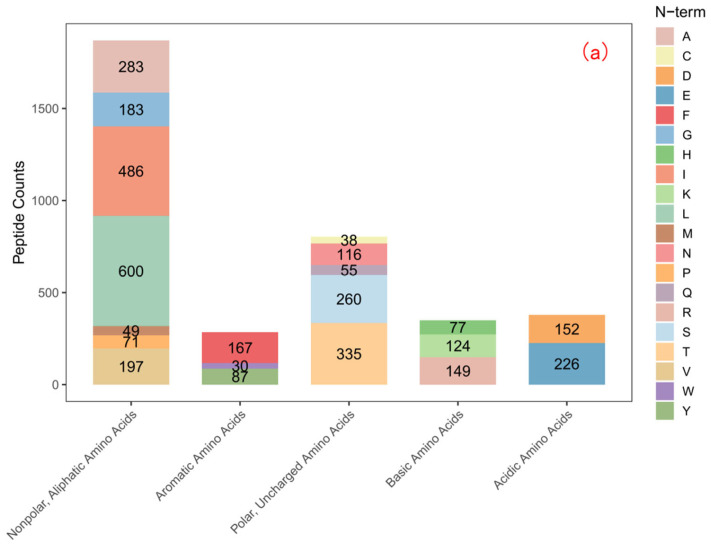

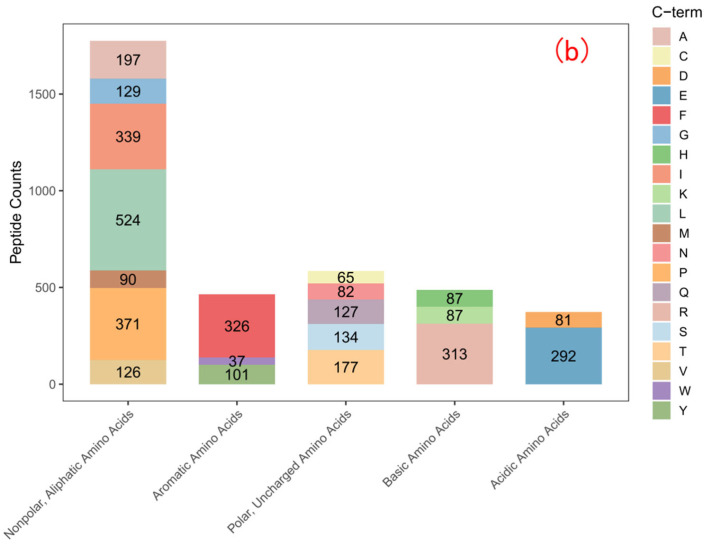
Amino acid frequency at the (**a**) N–terminus and (**b**) C–terminus of peptides identified in PZn.

Analysis of the amino acid frequency at the N– and C–termini of the PZn-constituting peptides reveals a clear structural motif crucial for zinc binding. The data (Figure 1) show a significant enrichment of acidic amino acids, particularly glutamic acid (Glu) and aspartic acid (Asp), at both termini, with an exceptionally high propensity at the C–terminus.

The C–terminus of a peptide, with its free α-carboxyl group, is a classic and high-affinity site for metal ion coordination. The profound enrichment of Asp and Glu at the C–terminus of PZn peptides is therefore highly significant. The deprotonated carboxylate groups (–COO^–^) in the side chains of these residues are optimal ligands for Zn^2+^. This finding provides direct and unambiguous evidence at the sequence level that confirms the role of acidic amino acids inferred from our bulk amino acid analysis (Section 3.3). It explains why these residues are critical for chelation: they provide the primary anionic coordination sites that bind the cationic zinc. Furthermore, the enrichment of acidic amino acids at the termini is consistent with the “terminal chelation model” proposed by Zhu et al. [22], where metal ions preferentially bind to the N– and C–terminal residues of peptides due to reduced steric hindrance.

#### 3.2.3. Stability, Allergenicity, and Toxicity Profile

The translation of a peptide–metal complex into a functional ingredient necessitates a preliminary safety and stability assessment. The predicted properties of partial dominant peptide sequences in PZn are summarized in Table 2.

To further verify the known functions of these representative peptides, we performed sequence alignment in the BIOPEP-UWM database, and no fully matched reported peptide sequences were retrieved. The instability index prediction shows a range of values. While some peptides are stable (e.g., Sequence 4, Index = 32.8), others are predicted to be unstable. It is crucial to note that these predictions are for isolated peptides in solution. In the context of the PZn complex, chelation with zinc can form stable coordination bonds that may significantly enhance the overall structural stability, potentially protecting labile sequences from degradation. More importantly, the vast majority of the dominant peptide sequences are predicted to be non-allergenic and non-toxic. This is a promising indicator for the safety of the PZn complex and supports its potential for further development in food or nutraceutical applications, pending necessary in vivo validation.

#### 3.2.4. In Silico Bioactivity Prediction

The bioactivity prediction for the peptide ensemble within PZn reveals a remarkable and diverse functional potential, as quantified in Table 3. A strikingly high number of peptides are predicted to possess potent antioxidant activities, with counts of 2669 for DPPH, 2546 for ABTS, 2818 for FRAP, and 2517 for ORAC scavenging.

This robust prediction is of paramount importance. It demonstrates that the zinc chelation process selectively enriches peptides that are intrinsically highly bioactive, particularly as antioxidants. This provides a molecular-scale rationale for the experimentally observed superior antioxidant activity of PZn over PP (Section 3.8). The enhancement is not solely due to the presence of zinc but is also rooted in the innate capacity of the selected peptide portfolio. The key residues for chelation (Glu, Asp) are often the same ones that act as potent electron donors in antioxidant mechanisms. Beyond antioxidants, the in silico predicted high counts for ACE-inhibitory (2714) and anti-inflammatory (3352) activities position PZn as a potential multifunctional ingredient, though these activities require further validation through in vitro and in vivo experiments. The umami taste prediction (1589 peptides) was performed using the UmamiTastePredict algorithm in the BIOPEP-UWM database (parameters: peptide length 2–10 aa, threshold 0.5), which is a widely used tool for predicting umami peptides [23].

### 3.3. Amino Acid Composition

The amino acid compositions of PP and PZn are presented in Table 4. Comparative analysis revealed significant changes in relative amino acid percentages after chelation. Notably, acidic amino acids (aspartic acid and glutamic acid), cysteine, and histidine showed increased proportions in PZn compared to PP. Specifically, aspartic acid increased from 3.14 ± 0.13% to 3.97 ± 0.11%, glutamic acid from 9.17 ± 0.19% to 9.37 ± 0.15%.

The chelating ability of peptides with zinc ions is closely related to the amino acid composition of peptides [24]. These compositional shifts suggest selective involvement of specific functional groups in zinc coordination. The increased proportion of acidic amino acids (Asp + Glu) from 12.31% (PP) to 13.34% (PZn) indicates that these residues are preferentially involved in zinc chelation, as their carboxyl groups (–COO^–^) are the primary ligands for Zn^2+^. The elevation of Cys (from 0.35% to 0.45%) suggests that thiol groups (–SH) also participate in coordination, forming stable Zn-S bonds. In contrast, the decreased proportion of hydrophobic amino acids (e.g., Leu, Ala) may be due to peptide aggregation induced by zinc chelation, which reduces the exposure of hydrophobic residues. These compositional changes further confirm the self-selective chelation mechanism of zinc ions with specific amino acid residues [22]. The apparent increase in these amino acids likely results from conformational changes during chelation that alter their accessibility or from selective incorporation of peptide fractions rich in these residues into the insoluble chelate complex.

### 3.4. Zeta Potential Analysis

Zeta potential measurements provided insights into the surface charge characteristics of PP and PZn (Table 5). PP exhibited a negative zeta potential of −16.70 ± 0.83 mV, reflecting the presence of negatively charged groups (e.g., carboxylate) on the peptide surfaces. After chelation, the zeta potential of PZn significantly increased to −9.23 ± 0.35 mV (*p* < 0.05), indicating partial neutralization of negative charges through coordination with positively charged zinc ions. Athira et al. [25] explored the Zeta potential of whey protein zinc-chelated peptides, and the results proved that the carboxyl group at the end of the peptide segment has a higher charge density, and the increase in net charge after chelation leads to an increase in Zeta potential. Concomitantly, the conductivity decreased substantially from 1.42 ± 0.03 mS/cm to 0.35 ± 0.001 mS/cm (*p* < 0.05). This reduction suggests decreased ionic mobility, possibly due to chelation-induced aggregation that reduces the number of free ions in solution [26]. The diminished absolute zeta potential value further supports the occurrence of structural reorganization and particle aggregation following zinc incorporation.

### 3.5. UV and FTIR Spectral Analysis

**Figure 2:**
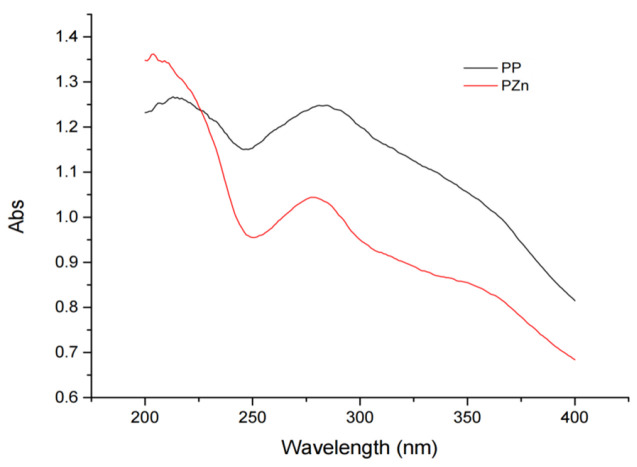
UV–VIS spectra of PP and PZn.

The ultraviolet absorption spectra of PP and PZn displayed notable differences (Figure 2). PP showed a characteristic absorption peak at 281 nm, attributable to π→π* transitions in peptide bonds and aromatic amino acids. After chelation, this peak underwent a blue shift to 277 nm with decreased intensity. This hypsochromic shift and hypochromic effect suggest alterations in the electronic environment of chromophores, likely resulting from coordination between zinc ions and functional groups such as carboxyl or amino groups in the peptides [27].

**Figure 3:**
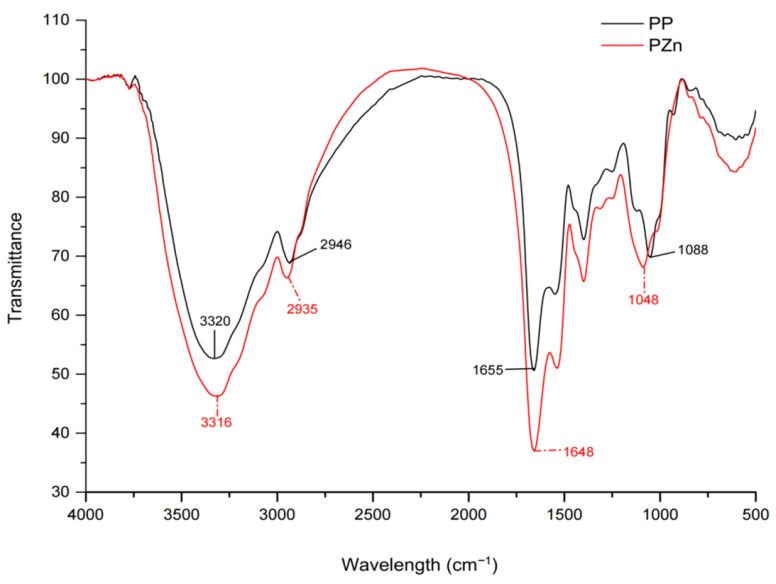
FT-IR spectra of PP and PZn.

FT-IR analysis provided further evidence of zinc-peptide interaction (Figure 3). Significant spectral shifts were observed in several key regions: The N-H stretching vibration (amide A band) shifted from 3320 cm^−1^ to 3316 cm^−1^; The C=O stretching vibration (amide I band) shifted from 1648 cm^−1^ to 1655 cm^−1^; The C-O stretching vibration shifted from 1048 cm^−1^ to 1088 cm^−1^; The amide III band (C-N stretching with N-H bending) shifted from 1401 cm^−1^ to 1535 cm^−1^. These shifts collectively indicate the involvement of carbonyl oxygen, carboxyl oxygen, and amino nitrogen atoms in zinc coordination [28]. The changes in hydrogen bonding patterns, particularly in the amide I and II regions, suggest that zinc chelation disrupts the original secondary structure of the peptides, leading to conformational rearrangements.

### 3.6. Circular Dichroism Analysis

**Figure 4:**
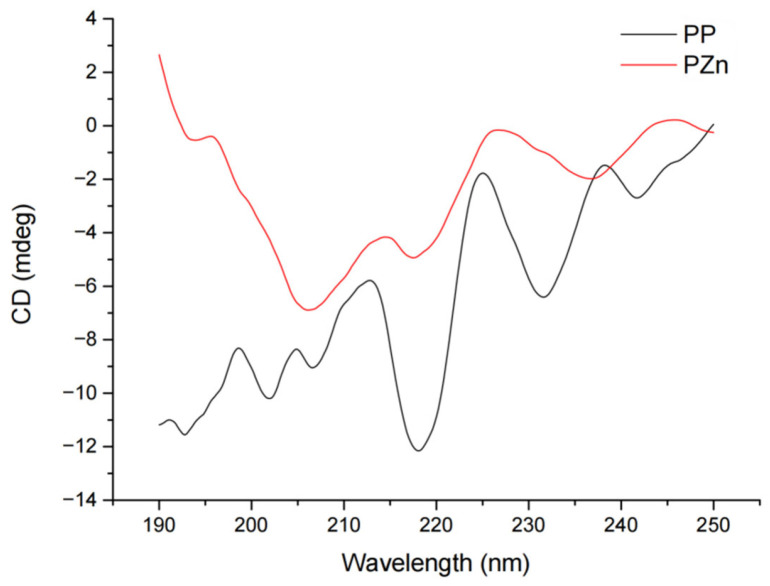
Circular Dichroic Spectrum of PP and PZn.

CD spectra revealed substantial changes in secondary structure following zinc chelation (Figure 4). Quantitative analysis using CDProWin7 software indicated significant alterations in structural composition (Table 6). The α-helix content decreased from 4.09 ± 0.11% in PP to 1.23 ± 0.05% in PZn (*p* < 0.01), while β-sheet content reduced from 41.81 ± 0.31% to 25.44 ± 0.25% (*p* < 0.01). Conversely, β-turn and random coil structures increased from 22.08 ± 0.27% to 27.37 ± 0.30% and from 32.35 ± 0.38% to 45.62 ± 0.16% (*p* < 0.05), respectively.

These structural transformations suggest that zinc coordination disrupts the native hydrogen bonding networks that stabilize ordered structures (α-helices and β-sheets) in the original peptides [29,30]. The consequent unfolding and structural loosening facilitate the formation of more flexible conformations (β-turns and random coils), which may enhance accessibility to functional groups involved in zinc binding and antioxidant activity.

### 3.7. SEM and EDS Analysis

**Figure 5:**
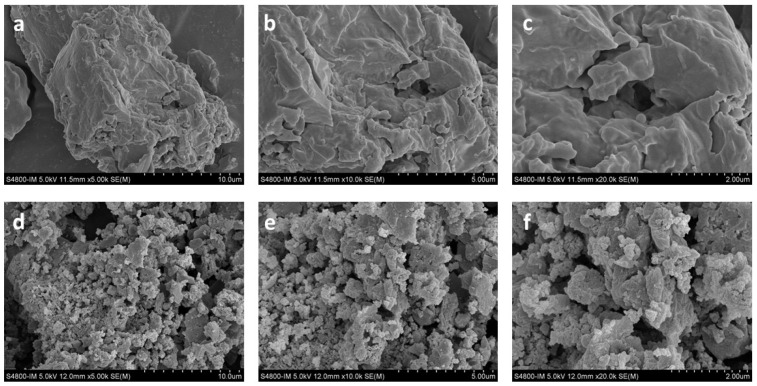
SEM images of PP (**a**–**c**) and PZn (**d**–**f**) at different magnifications (5000×, 10,000×, and 20,000×).

Scanning electron microscopy revealed distinct morphological differences between PP and PZn (Figure 5). PP presented as smooth, flaky particles with relatively homogeneous surfaces. In contrast, PZn exhibited rough, irregular surfaces with attached granular crystals, forming loosely aggregated structures. This morphological transformation likely results from intermolecular cross-linking mediated by zinc ions, which facilitates peptide aggregation through coordination bonds and electrostatic interactions [31].

The change in the microscopic morphology of the peptide–zinc chelate may result from the chelation reaction between the peptide and zinc ions, electrostatic interaction between the peptide and zinc ions, and the formation of ligand covalent bonds leading to granule formation. Metal ions can interact with peptides and significantly promote peptide aggregation [32]. The carboxyl, amino, and thiol groups on the peptide chain can form aggregates through hydrogen bonding with water molecules, and electrostatic interactions and intermolecular forces may contribute to the aggregation process [21].

**Figure 6:**
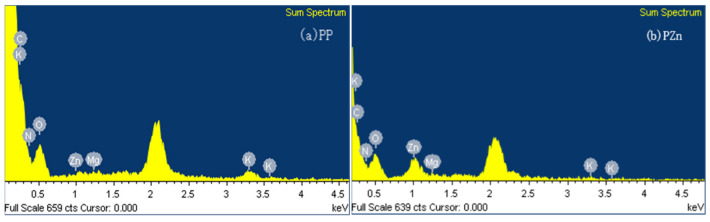
EDS spectra of (**a**) PP and (**b**) PZn.

Energy-dispersive X-ray spectroscopy confirmed the successful incorporation of zinc into the chelate complex (Figure 6). While PP showed only carbon, nitrogen, and oxygen peaks, PZn displayed a distinct zinc signal, with quantitative analysis indicating approximately 3.12% zinc content in the chelate. This elemental evidence unequivocally confirms the formation of peptide–zinc complexes.

### 3.8. Antioxidant Activity Evaluation

**Figure 7:**
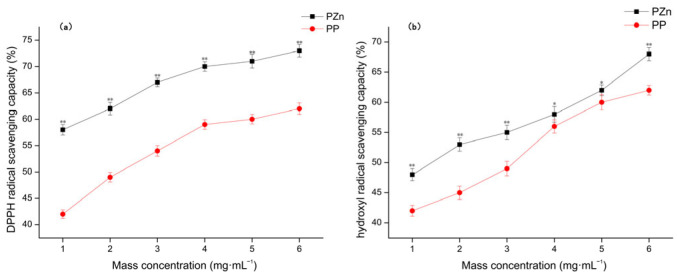
Antioxidant activity of PP and PZn. (**a**) DPPH radical scavenging capacity (**b**) hydroxyl radical scavenging capacity. Note: Data are presented as mean ± standard deviation (*n* = 3). * Significant difference vs. PP (*p* < 0.05); ** Highly significant difference vs. PP (*p* < 0.01).

The antioxidant capacities of PP and PZn were evaluated using DPPH and hydroxyl radical scavenging assays (Figure 7). Both samples demonstrated concentration-dependent radical scavenging activities. Notably, PZn exhibited significantly enhanced antioxidant capacity compared to PP across all tested concentrations.

In the DPPH assay, PZn showed approximately 25–40% higher scavenging activity than PP at equivalent concentrations. Similarly, in the hydroxyl radical scavenging assay, PZn demonstrated 20–35% superior performance. This enhancement can be attributed to several factors: (1) the electron-withdrawing effect of zinc may facilitate hydrogen donation from peptide functional groups; (2) the structural unfolding observed in CD analysis may expose additional antioxidant residues; and (3) zinc itself may participate in redox reactions that quench free radicals [33]. These findings align with previous studies reporting improved antioxidant activity in peptide–metal chelates compared to their native counterparts.

## 4. Conclusions

This study successfully prepared a pine nut peptide–zinc chelate (PZn) with a high chelation rate of 60.18 ± 1.77%. For the first time, the molecular composition of PZn was elucidated through peptidomic analysis, revealing that it is composed of a select group of low-molecular-weight peptides enriched in aspartic acid, glutamic acid, and cysteine. This finding demonstrates that zinc chelation is a self-selective process. The specific sequences identified provided a molecular basis for explaining the macroscopic structural changes observed by FT-IR, CD, Zeta potential, and SEM, establishing a clear chain of evidence from sequence to structure.

Furthermore, bioactivity prediction and experimental validation confirmed that the structural remodeling induced by zinc chelation directly contributes to a remarkable enhancement in antioxidant activity. The PZn complex exhibited superior radical scavenging capacities compared to the unchelated peptide mixture (PP). This work positions pine nut protein as a valuable raw material for developing a novel zinc supplement. Although this study is limited to the characterization of the complex as a whole, our future work will focus on the isolation and identification of specific peptide sequences within PZn with high chelation activity to further elucidate the structure–activity relationship at the molecular level. Subsequent studies should also prioritize the evaluation of in vivo bioavailability, safety profiles, and additional health-promoting activities of PZn in relevant biological models to fully realize its potential as a functional nutrient.

## Figures and Tables

**Table 1:** Molecular Weight Distribution of Peptides in PZn (*n* = 3).

Molecular Weight Range	Count	Percentage (%)
<1 kDa	2972 ± 54	80.65 ± 1.47
1–1.5 kDa	460 ± 21	12.48 ± 0.57
1.5–3 kDa	250 ± 16	6.78 ± 0.43
3–5 kDa	3 ± 2	0.08 ± 0.05
>5 kDa	0 ± 0	0 ± 0
Total	3685 ± 0	100 ± 0

**Table 2:** Property Prediction of Representative PZn Peptides.

No.	Sequence		Instability Index	Allergenicity	Toxicity
1	SERRGEEEDEDSSQKVRR	Ser-Glu-Arg-Arg-Gly-Glu-Glu-Glu-Asp-Glu-Asp-Ser-Ser-Gln-Lys-Val-Arg-Arg	159.89	Possible	No
2	NLRNQKPDFENDNGRFT	Asn-Leu-Arg-Asn-Gln-Lys-Pro-Asp-Phe-Glu-Asn-Asp-Asn-Gly-Arg-Phe-Thr	1.42	Possible	No
3	ERRGEEEDEDSSQKVR	Glu-Arg-Arg-Gly-Glu-Glu-Glu-Asp-Glu-Asp-Ser-Ser-Gln-Lys-Val-Arg	130.79	Possible	No
4	HNADNPEDADVYVR	His-Asn-Ala-Asp-Asn-Pro-Glu-Asp-Ala-Asp-Val-Tyr-Val-Arg	32.80	No	No
5	NLRNQKPDFENDNGR	Asn-Leu-Arg-Asn-Gln-Lys-Pro-Asp-Phe-Glu-Asn-Asp-Asn-Gly-Arg	0.27	Possible	No

**Table 3:** Predicted Bioactivity Profile of PZn-constituting Peptides.

Predicted Activity	Type	Count
Antioxidant	DPPH Scavenging	2669
	ABTS Scavenging	2546
	FRAP	2818
	ORAC	2517
Other Bioactivities	ACE Inhibition	2714
	Anti-inflammatory	3352
	Umami Taste	1589

**Table 4:** Amino acid composition of PP and PZn (%) (*n* = 3).

Classification	Amino Acid	PP	PZn
Acidic	Asp	3.14 ± 0.13	3.97 ± 0.11 **
	Glu	9.17 ± 0.19	9.37 ± 0.15 **
Basic	Lys	1.63 ± 0.17	1.06 ± 0.12 *
	Arg	6.32 ± 0.15	6.04 ± 0.12
	His	0.88 ± 0.08	0.97 ± 0.09
Neutral	Cys	0.35 ± 0.03	0.45 ± 0.05 *
	Ser	2.33 ± 0.09	1.60 ± 0.13
	Thr	1.30 ± 0.11	0.78 ± 0.10 *
	Gly	1.83 ± 0.09	1.36 ± 0.08 *
	Tyr	1.52 ± 0.04	0.84 ± 0.07
Hydrophobic	Pro	1.99 ± 0.14	1.46 ± 0.11 **
	Ala	1.95 ± 0.05	1.03 ± 0.02
	Val	1.92 ± 0.06	1.12 ± 0.06 *
	Met	0.65 ± 0.07	0.44 ± 0.09
	Ile	1.54 ± 0.13	0.90 ± 0.15
	Leu	2.85 ± 0.12	1.60 ± 0.16 **
Aromatic	Phe	1.59 ± 0.08	0.84 ± 0.02

Note: * Significant difference vs. PP (*p* < 0.05); ** Highly significant difference vs. PP (*p* < 0.01).

**Table 5:** Zeta potential and conductivity of PP and PZn (*n* = 3).

Sample	Zeta Potential (mv)	Conductivity (mS/cm)
PP	−16.70 ± 0.83	1.42 ± 0.03
PZn	−9.23 ± 0.35 *	0.35 ± 0.01 *

Note: * Significant difference vs. PP (*p* < 0.05).

**Table 6:** Secondary structure content of PP and PZn (%) (*n* = 3).

Sample	α-Helix	β-Fold	β-Turn	Random Coil
PP	4.09 ± 0.11	41.81 ± 0.31	22.08 ± 0.27	32.35 ± 0.38
PZn	1.23 ± 0.05 **	25.44 ± 0.25 **	27.37 ± 0.30	45.62 ± 0.16 *

Note: * Significant difference vs. PP (*p* < 0.05); ** Highly significant difference vs. PP (*p* < 0.01).

## Data Availability

The original contributions presented in this study are included in the article. Further inquiries can be directed to the corresponding authors.
